# MEG signatures of long-term effects of agreement and disagreement with the majority

**DOI:** 10.1038/s41598-021-82670-x

**Published:** 2021-02-08

**Authors:** A. Gorin, V. Klucharev, A. Ossadtchi, I. Zubarev, V. Moiseeva, A. Shestakova

**Affiliations:** 1 International Laboratory of Social Neurobiology, Institute of Cognitive Neuroscience, National Research University Higher School of Economics, Moscow, Russia; 2 Centre for Bioelectric Interfaces, Institute of Cognitive Neuroscience, National Research University Higher School of Economics, Moscow, Russia; 3 Department of Neuroscience and Biomedical Engineering, Aalto University, Espoo, Finland

**Keywords:** Cognitive neuroscience, Social neuroscience

## Abstract

People often change their beliefs by succumbing to an opinion of others. Such changes are often referred to as effects of social influence. While some previous studies have focused on the reinforcement learning mechanisms of social influence or on its internalization, others have reported evidence of changes in sensory processing evoked by social influence of peer groups. In this study, we used magnetoencephalographic (MEG) source imaging to further investigate the long-term effects of agreement and disagreement with the peer group. The study was composed of two sessions. During the first session, participants rated the trustworthiness of faces and subsequently learned group rating of each face. In the first session, a neural marker of an immediate mismatch between individual and group opinions was found in the posterior cingulate cortex, an area involved in conflict-monitoring and reinforcement learning. To identify the neural correlates of the long-lasting effect of the group opinion, we analysed MEG activity while participants rated faces during the second session. We found MEG traces of past disagreement or agreement with the peers at the parietal cortices 230 ms after the face onset. The neural activity of the superior parietal lobule, intraparietal sulcus, and precuneus was significantly stronger when the participant’s rating had previously differed from the ratings of the peers. The early MEG correlates of disagreement with the majority were followed by activity in the orbitofrontal cortex 320 ms after the face onset. Altogether, the results reveal the temporal dynamics of the neural mechanism of long-term effects of disagreement with the peer group: early signatures of modified face processing were followed by later markers of long-term social influence on the valuation process at the ventromedial prefrontal cortex.

## Introduction

Human behaviour is affected by the behaviour of others to a great extent. Conformal behaviour when individuals change their behaviours or beliefs in line with others’ behaviours or beliefs^1^, is associated with a number of forms of social influence, e.g. informational (when people looks for the group for guidance) or normative (when people desire approval of the group). Since a pioneering study of conformity by Solomon Asch in 1956^2^, where participants changed their opinion in line with the experimenter’s confederates despite their erroneous response, the impact of social influence on human behaviour became evident not only for professionals in the field of social science^1^, but also for a wider audience including policy makers.

In this study we continue the line of neuroimaging studies of neural substrate of social conformity^3^. Our group^4,5^ and other neuroimaging studies^6^ found that social conformity is mediated by reinforcement learning mechanisms and continuous performance monitoring^7^. Importantly, the involvement of ubiquitous mechanism of prediction-error mechanis ^8^ mediated by the dopaminergic neural circuitry^3,9,10^ seems to be implicated in neurobiology of social influence regardless whether it is normative or informational. Moreover, even when individuals want to avoid internal conflicts and preserve consistency in their attitudes, similar neural mechanisms of error-performance monitoring seem to be engaged^6^.

A number of functional magnetic resonance imaging (fMRI) studies have confirmed that opinions of others that disagree with one’s own opinions modulate activity of the posterior medial frontal cortex (pMFC), including the anterior part of the cingulate cortex (ACC), the insula, and the ventral striatum^3,11–15^. Moreover, electroencephalographic (EEG) studies have demonstrated differential processing of the opinions of others in terms of whether they agree or disagree with one’s own opinion. More specifically, the effect was manifested in the P200 visual evoked response and was allowed to suggest that to-be-rated stimuli, which had previously been judged similarly with the majority, could attract more perceptual attention^9^. In addition to the P200, a memory recollection associate component known as late positive complex (LPC) peaking at about 600–800 ms after the feedback was modulated by disagreement with the majority^16^. The conflict of individual and group opinions is often manifested in the feedback-related evoked response, also referred to as feedback-related negativity or FRN^17^, which has been interpreted as a neural correlate of general reinforcement learning^18^.

One of the first fMRI studies of conformity revealed that social influence modulated neural activity at the occipital and parietal cortices during a mental rotation task^12^. This finding was among very first to indicate the private acceptance of peers’ opinions through the modification of sensory processing. A later fMRI study^19^ specifically investigated the long-term effects of social influence on the neural activity underlying valuation of the stimuli. The exposure to their peers’ opinions affected the participants’ neural representations of the values assigned to faces in the ventral striatum and the OFC. During the second presentation of faces, activity in the OFC and the ventral striatum increased for faces that the group previously rated more favourably than the individual did, as compared to faces that the group rated less favourably. The authors interpreted their finding in light of the true modifications of beliefs and opinions evoked by social influence. This finding supports the concept of social influence emphasizes the rewarding value of social approval^1^.

The question as to whether the observed conformal changes of normative behaviour is driven by outward compliance or by the strive to fit peers’ expectations^13,20^ is still debated. Moreover, the majority of neurocognitive studies regarding social deviance focused on the neural signatures of immediate exposure to group feedback^6,9,13,20,21^. Up to date, only a few studies attempted to investigate the neural correlates of the long-term effects of social influence, e.g. changes in the stimuli processing after previously experienced social interaction associated with them^19,22^. Some of them found evidence of long-lasting memory alterations induced by the erroneous group recollections were predicted by enhanced amygdala activity^22^, whereas others observed conformal alteration of the values assigned to the stimuli engaged the orbitofrontal cortex (OFC) and nucleus accumbens^19^.

To date, both theories of private acceptance^19,22^ and public compliance^13^ are supported by, primarily, fMRI evidences^6^. It is difficult to model pure motivation of outward behaviour or true preference change in the laboratory conditions. Moreover, depending on the specific experimental demands, different neurocognitive functions can be associated with changes of preferences or believes: memory, attention, valuation, emotional processing. That is why adding temporal dimension to the spatial mapping may help to reconcile different findings of neurobiology of social influence and possibly offers a novel theoretical framework. During the last few years, the results of earlier fMRI studies^3,13,14,19,23^ of social influence have been complemented with magneto- and electroencephalography (M/EEG) findings^9,24,25^, which have much better time resolution. For example, the recent MEG study^25^ revealed neural signatures of disagreement with the majority (in the 220–320 and 380–530 ms time windows) in the ventromedial prefrontal cortex (VMPFC) and PCC, which differed from the previous fMRI findings^6^. Zubarev and colleagues also found that the disagreement with the majority increases frontal theta oscillations, which may prevent deviations from normative behavior (or group opinion)^25^.

Although the time-resolved M/EEG and spatially precise fMRI neuroimaging methods cannot be integrated in a straightforward manner^26^, they may critically complement one another giving precise information about temporal and spatial characteristics of the brain activity. That is why applying MEG to analyse the neurodynamics of cognitive processes may seem beneficial. In addition to its high temporal resolution as in EEG, MEG possesses fine spatial resolution for cortical sources^27^. Following this line of thinking, in this study, we used MEG to investigate the long-term effects of earlier disagreement with peers’ opinions on the neural processing of faces.

We used a modified version of the face judgment task^14^ to study the long-term effect of social influence. The study was composed of two sessions. During Session 1, participants rated a set of faces based on their trustworthiness. In each trial, the participant was informed how a large group of students from the same university (group rating) rated the face. This procedure allowed us to create the situation where participants' opinions either repeatedly matched or mismatched with the opinion of the group. After a 30-min break, in Session 2, they rated the same set of faces again, receiving no feedback. The MEG findings for Session 1 are reported in our previous study^25^, which showed, that conflicts with group’s opinion during Session 1 triggered a ‘reward prediction error’. Such perceived mismatch with group’s opinion activated the error-processing circuitry in the anterior and posterior medial cortices as indexed by the MEG activity. In the current study, we focus on the analysis of MEG signal in Session 2 hypothesizing that the group opinion about faces given in Session 1 could affect neural processing of the same faces later on.

## Results

### Behavioural results

In our study, the participants rated a set of faces depending on their trustworthiness. In Session 1, they received feedback with the group rating assigned to each stimulus, then, after a 30-min break, the subjects repeatedly rated the same set of stimuli with no group feedback (Session 2). Average initial rating during Session1 was 4.3 (± 0.67). During Session 2, the participants (a) adjusted their initial rating in line the group rating in 46% of trials, (b) repeated their initial rating in 28% of trials, and changed their initial rating in the opposite direction relative to the group rating in 26% of the trials. A two-way ANOVA revealed significant effect of *conflict direction* (group’s opinion is *more negative*, *similar* or *more positive* than subject’s initial option): F(1, 19) = 116.1, *p* = 0.00001. We further analyzed the effect of social influence using a subset of faces with intermediate initial ratings (4 and 5) to account for possible artificial correlations caused by repeated measurements and the distribution of initial ratings (if the initial rating was at the edge of the scale, the group feedback as well as follow-up rating could be the same or shift toward the center of the scale, therefore, it is difficult to distinguish true conformal change and an accidental approach to the group rating). The two-way ANOVA also showed a significant main effect of *conflict direction*: F(1, 19) = 12.54, *p* = 0.0007. Overall, participants significantly changed their initial ratings in line with the group ratings (for further details of the behavioural results, see Zubarev et al.^25^).

### MEG markers of agreement and disagreement with group opinion

**Figure 1:**
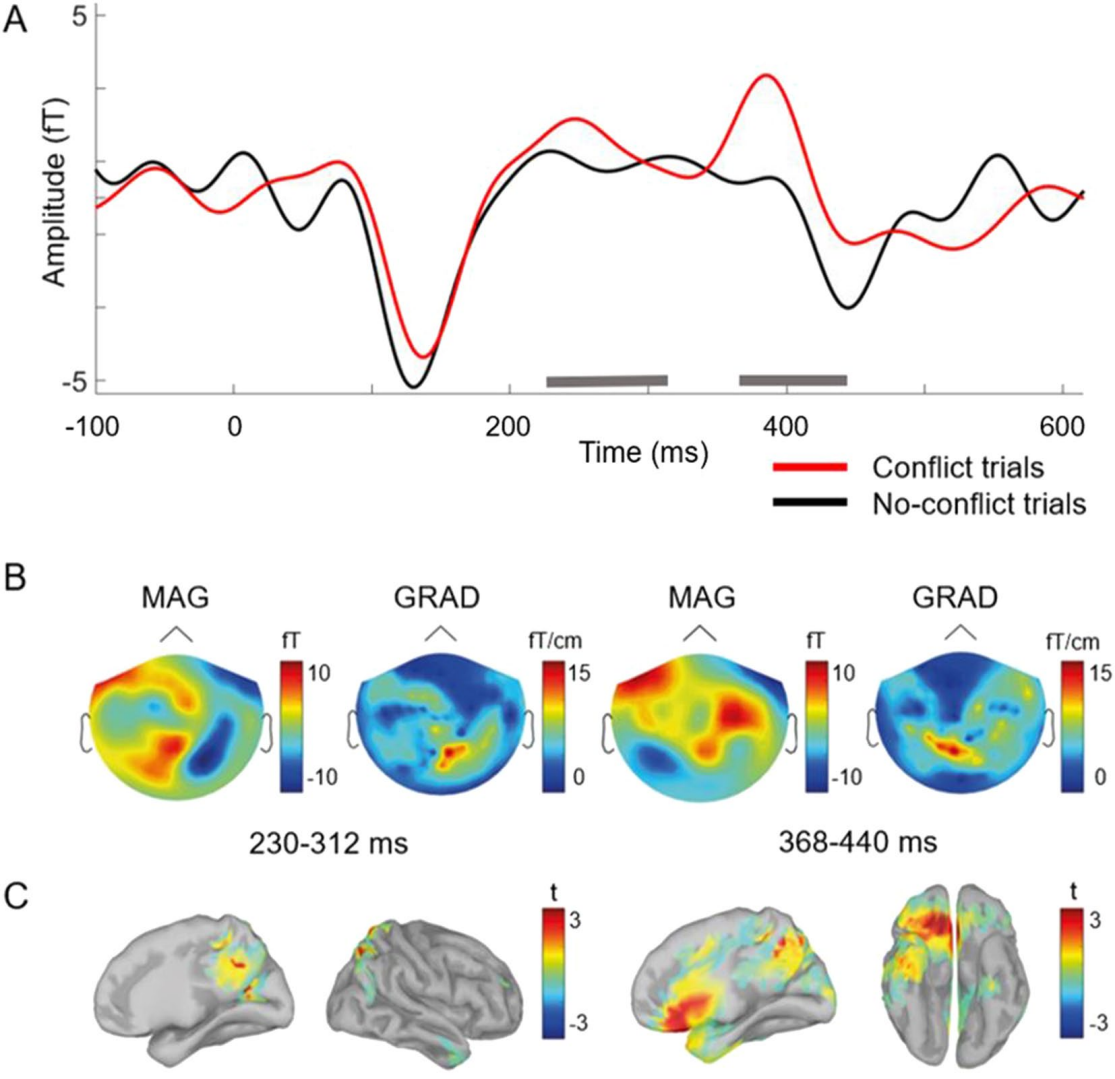
MEG signatures of the long-term effects of agreement and disagreement with the majority (Session 2). (**A**) Averaged ERF across magnetometers, black line: no-conflict trials, red line: conflict trials. Grey bars indicate the time intervals where significant differences were found between conflict and no-conflict trials. (**B**) MEG markers of agreement and disagreement with the majority in sensor space (ERF in conflict trials *minus* ERF in no-conflict trials). Averaged event-related field topographies for the magnetometers (MAG) and averaged norm topographies for the planar gradiometers (GRAD). (**C**) MEG markers of agreement and disagreement with the majority in the source space (t-statistics).

The gradiometer data analysis of the brain activity revealed two spatiotemporal clusters of brain activity that significantly differed between the conflict and no-conflict trials during Session 2 (Table 1). The conflict trials presented faces that were previously encoded (Session 1) in the context of group ratings that disagreed with the participant’s initial rating. The no-conflict trials presented faces that were presented previously (Session 1) in the context of group ratings that agreed with the participant’s initial rating. The first posterior cluster was associated with differences within 230–332 ms after the onset of the stimulus; the second frontal cluster exhibited differences in activity between 368 and 440 ms after the onset of the stimulus (Fig. 1A).

**Table 1:** Cluster statistics (in sensor space) for brain activity, which significantly differed between the conflict and no-conflict trials.

Sensor type	Time window (ms)	Size	Sign	*p* value (FDR corrected)
GRAD	230–332	557	negative	0.004
GRAD	368–440	182	negative	0.01
MAG	158–312	692	positive	0.004
MAG	220–340	460	negative	0.005

*GRAD* gradiometer sensor, *MAG* magnetometer sensor.

The magnetometer data analysis confirmed the presence of significant spatiotemporal clusters of the brain activity that differed in the conflict trials compared to the no-conflict trials (Table 1) in the earlier (158–312 ms) and the later (220–340 ms) time windows. The activity spotted by magnetometers started some 50 ms earlier as compared with gradiometers but its averaged. The grand-averaged differences in event-related fields (ERFs) (conflict *minus* no-conflict trials) for the 230–312 ms time window are presented in Fig. 1B.

All additional comparisons (conformal vs. non-conformal, peers-higher vs. peers-lower, peers-lower vs. no-conflict, and peers-higher vs. no-conflict) revealed no statistically significant differences in the MEG signals (p-value no less than 0.5). Overall, we found that peer ratings of facial trustworthiness during Session 1 triggered changes in the processing of faces that were manifested in the ERFs during Session 2. These traces of social influence on facial trustworthiness were observed within 158–440 ms after the face onset. Importantly, the neural markers of social influence were not related to a difference in average facial trustworthiness in the conflict trials when compared to the no-conflict trials: mean facial trustworthiness in conflict trials = 4.24 (SD = 0.77), in no-conflict trials = 4.22 (SD = 0.78), F (1, 38) = 0.01, *p* = 0.93. Therefore, observed differences in ERFs were not related to the face rating or conformal changes in behavior, probably being caused by the feedback during Session 1, and could be judged as traces of the previous conflict with normative group opinion.

### MEG markers of agreement and disagreement with group opinion

We constrained the source-space analysis to the time windows of the significant clusters revealed during the analysis of the sensor-space data. We found two significant clusters with differences in the conflict trials when compared to the no-conflict trials. The first cluster spread over the superior parietal lobule (SPL), intraparietal sulcus (IPS), precuneus, and cuneus within a 226–288 ms time window, while the second significant cluster was found within the 388–412 ms interval at the anterior cingulate cortex (ACC) and the OFC; t-maps of paired t-test revealed greater activity in the conflict trials (see Fig. 1C; Table 2). Thus, disagreements with the majority triggered long-lasting changes in the processing of faces at the parietal, cingulate, and orbitofrontal cortices.

**Table 2:** Cluster statistics in source space.

Hemisphere	Time window (ms)	Cluster mass	Sign	*p* value (FDR corrected)	Structures
Right	226–288	6615	positive	0.045	Superior parietal lobule, intraparietal sulcus, precuneus, cuneus
Right	388–412	2258	positive	0.045	Orbitofrontal cortex, anterior cingulate cortex

## Discussion

This is the first MEG study to explore the neural correlates of the long-term neural signatures of social influence. At the behavioural level, groups’ judgments of faces influenced participants’ judgments of facial trustworthiness. The participants changed their ratings in line with the group ratings. Based on this observation, one can hypothesize that face judgment task evoked the conformal changes in opinion of the participants similarly to the previous studies where subjects rated attractiveness of the faces on photos. To investigate long-term neural traces of group influence, we compared ERFs observed during Session 2 to faces for which group ratings of facial trustworthiness previously agreed or disagreed with the participant’s own ratings. At the neurophysiological level, we found that the previously experienced conflicts with group opinions were manifested in an enhanced neural response to faces within two time windows: 226–288 ms (IPS, SPL, and precuneus) and 318–412 ms (ACC and OFC) after face onset.

Interestingly, long-term MEG signatures of disagreement with the group opinion in the current study (Session 2) corresponded in latency with the conflict-related evoked responses (Session 1) reported in the previous MEG study^25^. Similar to the current study, group opinions that conflicted with individual opinions evoked stronger MEG responses in the precuneus and ACC. Moreover, our results well corroborate previous EEG studies of social influence on judging the attractiveness of faces^4,9^. They demonstrated the significant difference between evoked responses to group ratings of popularity (Session 1), which conflicted with participants’ rating (as compared to group ratings, which did not conflict with participants’ rating), also at a latency of around 200 ms. Thus, an explanation of the long-term MEG signatures of disagreements with the majority in our study could be proposed based on a performance monitoring mechanism of social influence^17,28–31^. We can speculate, that faces presented for the second time (Session 2) trigger cortical activity similar to the FRN responses (evoked by conflicting group ratings during Session 1) associated with reinforcement learning circuitry. In previous EEG studies, cues that predicted future losses evoked stronger ERN responses as compared to cues that predicted future rewards^32,33^. Furthermore, cues provided probabilistic information about future losses evoked stronger neural responses than cues predicting probable future rewards^17,30,31^. Importantly, the topography of the evoked responses produced by cues that predicted future losses coincided with the topography of the evoked responses produced by losses themselves. Therefore, faces for which participants previously disagreed with the majority could serve as cues predicting probable future “social” losses or conflicts.

Previous fMRI studies demonstrated elevated precuneus activity during disagreements with peers’ opinions about healthy and unhealthy foods^34^, facial attractiveness^3^, and facial trustworthiness^25^. Furthermore, the precuneus has been implicated in general error processing^35,36^. Accordingly, precuneus activity could encode long-term traces of social learning signals. Interestingly, the precuneus has been associated repeatedly with updating trustworthiness information in situations of inconsistency between past and present moral information provided to subjects^37,38^. Thus, alternatively, early parietal MEG correlates of social influence on facial trustworthiness may indicate that conflicts with group opinion evoke long-term changes in the processing of faces.

We also observed the early (226–288 ms) signatures of the long-term effects of conflicts with group opinion at other parietal cortices (IPS and SPL). According to the theory of top-down attention, the SPL belongs to memory retrieval circuitry^39^. Thus, the stronger activity of the SPL in the present study could indicate enhanced memory strength or the enhanced familiarity of faces induced by conflicts with group opinion^40^. It is also possible that previous disagreements with group opinion regarding facial trustworthiness may make these faces more salient and, therefore, affect the strength of their memory traces to facilitate future memory-guided social interactions. Although, we did not find a memory-related LPC activity associated with retrieval in Session 2, future studies could employ a separate test on recognition memory for previously disagreed as compared with agreed faces may shed light on neural processes involved in internalization of social influence provided the earlier finding of human tremendous and almost limitless memory for pictures over, e.g. other objects, such as words^41^.

The early IPS finding obtained in our MEG study well corroborates the fMRI results of the previous study of social influence, which demonstrated conformity-related activity of this region during a mental rotation task^12^. In the current study, the right IPS was particularly active if participants’ opinions previously disagreed with the peers’ opinions. Earlier studies also suggested that the IPS encompasses a system in which various quantities are represented according to a mental line^42^. Thus, the IPS activity observed in the present study could indicate a recalibration of the trustworthiness ratings evoked by peer influence. We speculate that the long-term signatures of social influence in IPS activity could further indicate an internalisation of group opinion.

We also documented the activity of the OFC and ACC in response to the previously disagreed-upon stimuli. These ventromedial correlates of disagreement with the majority may index internalisation of peer influence within the 388–412 ms interval at the OFC. This result corroborates Zaki et al.’s fMRI finding^19^ of the long-lasting effects of social influence on OFC activity obtained in a similar experimental task complementing it with the temporal information. Of note, the fMRI evidence of the involvement of the OFC in value-based learning, reward processing, decision-making, and social cognition is ample^43–49^. Therefore, this MEG finding of OFC and pMFC activations could be interpreted in the framework of theory of reward-prediction error^50^ and serve as additional neuroimaging proof, suggesting that exposure to social norms modulates participants’ neural representations of values assigned to stimuli.

We observed unspecific as to the direction of the conflict MEG correlates of disagreements with peers’ opinions at the OFC, as we found no statistically significant difference between MEG responses in peers-higher and peers-lower trials. Few methodological differences can explain such discrepancies between the earlier fMRI study and our MEG findings. First, the statistical analysis in MEG studies requires more trials than in fMRI studies, which can lead to a smaller statistical power in our study. Second, the experimental instructions varied between the studies: participants in our study rated facial trustworthiness, while facial attractiveness was rated in the previous study^19^. Obviously, more studies are needed to clarify this discrepancy.

Importantly, our previous MEG results^25^ showed activation of VMPFC and PCC around 220–350 ms to immediate conflict of opinions in Session 1, which corroborated the previously found error-related EEG correlates of conflict of opinions^4,9^. Of note, all three studies replicated feedback-related negativity brain responses following the perceived opinion discrepancy. Here, we further compared our MEG results with the previous EEG finding in Session 2 in search for the long-lasting effects of perceived conflict of opinions. Similar to the previous EEG study^9^, we found no significant long-term effect of disagreement with the peers’ opinions on the largest face-specific M170 component in Session 2, which has been implicated in early stages of multiple-level facial perception^51^. In the EEG study of Schnuerch and Gibbons^9^, the analysis of the evoked responses revealed a stronger evoked response (posterior P2) between 155 and 175 ms to faces for which participants previously agreed with the majority, as compared to those on which they disagreed. In Session 2, however, we found a significant magnetometer cluster in sensor space in 158–312 ms time window. Based on this observation, one can hypothesize that the earlier MEG signature of previous conflicts with the majority may be the equivalent to P2 in EEG studies. This sensor space finding supports hypothesis that conflict-trials evoke enhanced attention. Although in the source space we do not see a significant cluster at the time window corresponding to the P2 window, the hypothesis of conflict-related attention changes is indirectly supported by the earlier medial parietal MEG cluster^52^. As was shown by another MEG study of faces^53^, it may be difficult to compare MEG and EEG studies without a simultaneous recording, as different sources could be spotted by EEG and MEG^27^. A concurrent EEG/MEG recording would be necessary to perform in order to elucidate long-term effects of social influence of private acceptance is mediated via attention that in its turn is marked by P2.

Unlike in our previous EEG^4^ and fMRI^3^, we did not find statistically significant proof for the long-term neural correlates of conformal adjustments of ratings following disagreement with the majority. This could be explained by several reasons: (1) the sample was unbalanced since the proportion of conform to no-conform trials varied across the subjects; (2) to control the regression to the mean, we excluded a large number of trials, which could lead to a low signal-to-noise ratio. Further MEG studies using different behavioural paradigms or a larger number of trials are needed to find the long-term memory effects of conformity to the majority. To extend our knowledge on neural mechanisms of social influence, it is necessary to study domains other than facial trustworthiness or attractiveness, using stimuli other than faces. A single domain finding is clearly a limitation of our study. Furthermore, it would be of tremendous importance to extend studies of neurobiological mechanisms of social influence into other domains of interest to policy-makers.

In summary, the present MEG study indicates that (dis)agreements with majority opinion induce changes in the processing of faces. First, the history of disagreements with majority opinion on facial trustworthiness can be traced in MEG activity in the two latency windows: 226–288 ms (the IPS, SPL, and precuneus) and 318–412 ms (the ACC and the OFC) after face onset. The earlier traces of disagreements with the majority in the right parietal lobe may indicate modulation of the social processing of faces, as well as modulation of facial memory. Second, these early signatures of modified face processing are followed by later markers of long-term social influence on the valuation process at the ventromedial prefrontal cortex. Taken together the aforementioned results add novel spatiotemporal details of the neural mechanisms of social influence. Our results integrate in time and space the earlier perceptual/attentional post-effects of disagreements with majority opinion at the medial parietal cortices with the subsequent effects at the prefrontal cortex associated with performance monitoring and valuation. Although an additional study with a large sample size would be necessary to further support our findings of the spatio-temporal signatures of long-term effects of conformal changes, we believe that these MEG findings of long-term traces of disagreement with majority opinion remains a significant complement previous fMRI and EEG findings and motivates further investigation of neural circuitry of social influence.

## Methods

### Participants

Twenty female volunteers participated in the experiment (mean age 24.2 years; range 18–28 years; right-handed; with normal or corrected eyesight). All participants reported no history of neurological or psychiatric disease, drug abuse, or head trauma. The data for one participant were discarded from the group analysis due to a large number of artefacts. For taking part in the experiment, participants received monetary compensation of 500 roubles (the equivalent of USD 16), which typically covered one day’s food (grocery store) expenses for a single person in Moscow.

All participants were familiarised with the experimental procedure and signed the informed consent form. We tested the participants’ personality traits using the Eysenck Personality Inventory^54^, the Sensation Seeking Scale^55^, a short version of the Big Five questionnaire^56^, the Mehrabian Conformity Scale^57^, individual levels of anxiety^58,59^, and the Locus of Control questionnaire^60^. We found no significant correlations between the behavioural results and the personality traits identified using these tests and guidelines (*p* > 0.2).

### Experimental procedure

**Figure 2:**
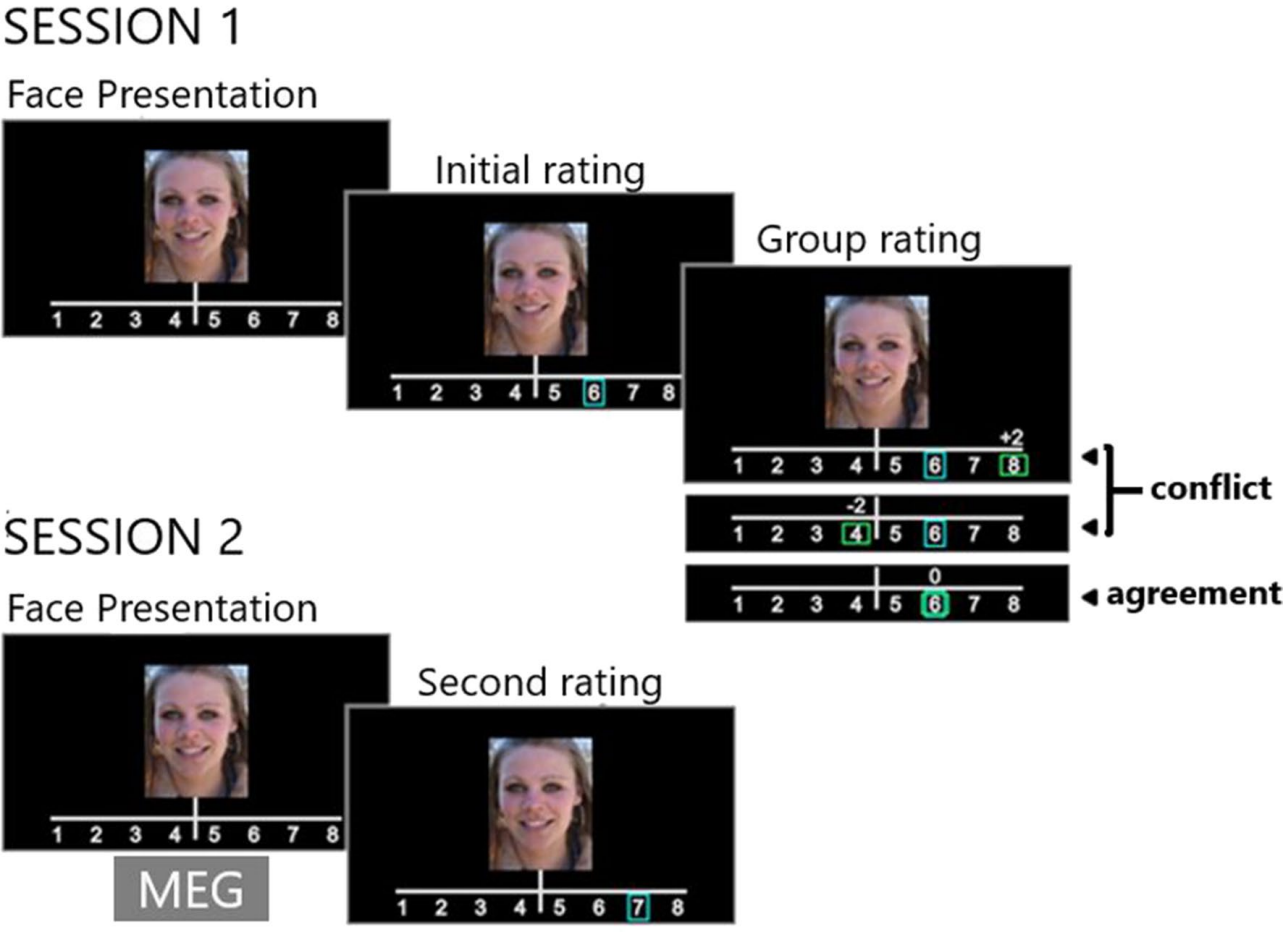
Experimental design. During Session 1, participants rated the trustworthiness of faces on a scale from 1 to 8. Next, participants learned the group rating for that face, which could either agree (no-conflict trials) or disagree (conflict trials) with the participants’ initial rating. In order to identify the long-term effects of social influence, the recorded MEG events were locked to the second face presentation.

In the present study, we used a modified version of the face-judgment task^14^ to investigate the long-term effects of social influence on the processing of faces. During the first part of the experiment (Session 1), the participants were presented with 222 photographs of female faces (stimulus duration 2000 ms; intertrial interval = 2500–3000 ms; overall session duration = 35 min; see Fig. 2).

Participants were instructed to decide whether to entrust the person viewed onscreen with a substantial sum of money (the equivalent of USD 1500) in a hypothetical situation. They rated each face using an eight-point scale (1 = very untrustworthy; 8 = very trustworthy). Each participant’s rating (the initial rating) was indicated on the screen by a blue rectangular frame immediately after the button press. Next, the participant was informed of how a large group of students from the same Russian university had rated the same face (the group rating). The group rating was indicated by a green rectangular frame. The difference between the participant’s initial rating and the group rating was displayed above the scale (0, ± 2, or ± 3 points). Group ratings were generated by the pseudorandom algorithm to maintain the proportion of conflict (peers-higher, peers-lower) and no-conflict trials. Each trial type (conflict trials/peers-higher; conflict trials/peers-lower; no-conflict trials) was presented 74 times. Participants believed that the group ratings were averaged based on previously collected data (for further details on the paradigm and analysis of the behaviour and MEG data during Session 1, see Zubarev et al.^25^).

After a 30-min break, participants attended the second part of the experiment. They rated the same set of faces again during the MEG recording (second rating, Session 2). The participants were not informed in advance that they would rate the faces twice. We focused on the brain activity during Session 2 to detect traces of the conflicts (disagreements with the majority) that participants experienced during Session 1. The ERFs were locked to the second presentation of faces during Session 2.

In the conflict trials, each participant was exposed to faces that were presented previously (Session 1) in the context of group ratings that disagreed with the participant’s initial rating. In the no-conflict trials, each participant processed faces that were presented previously (Session 1) in the context of group ratings that agreed with the participant’s initial rating. Additionally, we calculated ERFs to faces that had been presented previously in the context of group ratings that were lower than the participant’s initial rating (peers-higher trials) or in the context of group ratings that were higher than the participant’s initial rating (peers-lower trials). To trace the brain activity related to the behavioural conformal changes, we split conflict trials into conformal (the second rating was closer to the group rating than the first) and non-conformal (the rest of the trials) groups, depending on the subsequent changes in individual ratings.

The debriefing indicated that the participants remembered 10 to 20 faces presented during the experimental sessions, but the participants were unable to recall their own initial ratings. Importantly, each face was presented only once per experimental session.

### Data collection and preprocessing

Magnetic fields were measured using a 306-channel (102 magnetometers, 204 planar gradiometers) whole-head Elekta Neuromag VectorView MEG scanner (Elekta AB, Stockholm, Sweden) with a sampling rate of 1000 Hz. The data were recorded continuously with the application of band-pass filtering from 0.1 to 333 Hz. To minimize the influence of external sources, we used the temporally extended source signal separation (tSSS) method^61^ after the data acquisition. Head movements were corrected to the default head coordinates using signals from four head position indicator (HPI) coils^62^ placed in the areas of the F3 and F4 electrodes and bilaterally on the mastoids. The procedures were performed using the MaxFilter software provided by the Elekta Neuromag Company.

Further, we processed the data using the Brainstorm toolbox^63^. All of the recordings were inspected manually for artefacts. Eye blinks and heartbeats were detected automatically from the electrocardiographic and electrooculographic data using the Brainstorm artefact detection functions. Eye blink and cardiac artefacts were suppressed using the Brainstorm implementation of the signal-space projection method^64^.

For efficient co-registration of the MEG and MRI data, we used the Polhemus Fastrak motion tracker (polhemus.com) to digitise three anatomical points (two preauricular points and the nasion), HPI coils, and 100 additional points on the scalp.

### Analysis of ERFs

MEG data was divided into epochs (from –200 to 800 ms) centred on the onset of the face presentation (t = 0) and then down sampled to 500 Hz to optimise the signal processing time. A zero-order polynomial detrend based on the prestimulus interval (from –200 to 0 ms) was applied to each trial to remove the direct current offset. ERFs were calculated for each trial type.

To compare the ERFs in the no-conflict and conflict trials, we used the Brainstorm interface of the Fieldtrip toolbox^65^ and performed a paired spatiotemporal cluster-corrected permutation test (the cluster inclusion threshold was set to *p* < 0.01 with 1000 permutations over the full epoch time windows). The cluster *p* values were defined as the probability of observing the cluster with the higher mass, separately for the positive and negative clusters. The test was calculated separately for the magnetometers and the gradiometers. We used the same statistical analysis to compare the peers-higher, peers-lower, and no-conflict trials and to compare conformal and non-conformal trials.

### Head modelling

We collected individual T1-weighted MRIs for all subjects using a 1.5 T Siemens scanner and constructed individual 3-D brain models with the FreeSurfer software toolbox^66^. These models were imported into the Brainstorm workspace. Forward solutions for the individual head models were calculated using the overlapping spheres approach.

### Source analysis

We used the Brainstorm implementation of the depth-weighted minimum norm estimation algorithm^27^ with an unconstrained orientation to find the cortical current density distribution underlying the observed ERFs. The norms of the moments of the two locally tangential dipoles were then computed for each vertex and projected from the individual models onto the default anatomy model (6000 vertices) using the iterative closest neighbour search algorithm, as implemented in the Brainstorm software.

To maximize the test power and resolve statistical activations in space, we performed statistical analysis of the source-space data within the time windows, where significant activations were detected based on the sensor-space data. A similar approach has been used in recent MEG studies^67–69^. The paired spatiotemporal cluster-corrected permutation test with a cluster inclusion threshold of *p* < 0.01 (uncorrected *p* value, two-tailed test) was performed to compare the conditions. The cluster mass was defined as the sum of the signed t-scores of the vertices within the contiguous space–time region. The cluster *p* value was estimated as the fraction of the times the cluster of a larger mass was observed over 1000 random permutations^25,70^.

### Ethical approval

The study was approved by local ethics committee of National Research University Higher School of Economics and was performed according to relevant regulations. All subjects read and signed an informed consent prior to the experiment, and received a monetary reward as compensation for participation.

### Informed consent

All participants were familiarised with the experimental procedure and signed the informed consent form.

## Supplementary Information

Supplementary Information.

## Data Availability

The datasets analysed during the current study are available from the corresponding author on reasonable request.
