# Author Correction: MEG signatures of long-term effects of agreement and disagreement with the majority

**DOI:** 10.1038/s41598-021-88176-w

**Published:** 2021-04-19

**Authors:** A. Gorin, V. Klucharev, A. Ossadtchi, I. Zubarev, V. Moiseeva, A. Shestakova

**Affiliations:** 1 International Laboratory of Social Neurobiology, Institute of Cognitive Neuroscience, National Research University Higher School of Economics, Moscow, Russia; 2 Centre for Bioelectric Interfaces, Institute of Cognitive Neuroscience, National Research University Higher School of Economics, Moscow, Russia; 3 Department of Neuroscience and Biomedical Engineering, Aalto University, Espoo, Finland

Correction to:* Scientific Reports* 10.1038/s41598-021-82670-x, published online 8 February 2021

The original version of this Article contained errors in the Affiliations.

Affiliation 1 was incorrectly given as “Centre for Cognition and Decision Making, National Research University Higher School of Economics, 20, Myasnitskaya Street, 101000, Moscow, Russian Federation.”

The correct affiliation is listed below:

International Laboratory of Social Neurobiology, Institute of Cognitive Neuroscience, National Research University Higher School of Economics, Moscow, Russia.

Additionally, an affiliation for author A. Ossadtchl was omitted. The correct affiliation is listed below as affiliation 2.

Centre for Bioelectric Interfaces, Institute of Cognitive Neuroscience, National Research University Higher School of Economics, Moscow, Russia.

The original Article and accompanying Supplementary Information file has been corrected. The corrected Supplementary Information file is also linked to this correction notice.

## Supplementary Information

Supplementary Information

