# Supplementary material for: MEG signatures of long-term effects of agreement and disagreement with the majority
Source: Sci Rep. 2021 Feb 8;11:3297. doi: 10.1038/s41598-021-82670-x (PMC7870674; doi:10.1038/s41598-021-82670-x)
Supplement: Supplementary file 1 — Supplementary Information. [file 41598_2021_82670_MOESM1_ESM.docx]

**MEG signatures of long-term effects of agreement and disagreement with the majority**

Supplementary materials

Gorin A.^1^, Klucharev V.^1^, Ossadtchi A.^2^, Zubarev I.^3^, Moiseeva V.^1^ and Shestakova A.^1^

^1^ International Laboratory of Social Neurobiology, Institute of Cognitive Neuroscience, National Research University Higher School of Economics, Moscow, Russia

^2^ Centre for Bioelectric Interfaces, Institute of Cognitive Neuroscience, National Research University Higher School of Economics, Moscow, Russia

^3^ Department of Neuroscience and Biomedical Engineering, Aalto University, Espoo, Finland

Corresponding author: Aleksei Gorin, Centre for Cognition and Decision Making,

National Research University, Higher School of Economics, Moscow, Russia

e-mail: [agorin@hse.ru](mailto:agorin@hse.ru)

phone: +7 977 408 3770
address: 20, Myasnitskaya Street, Moscow, Russia, 101000


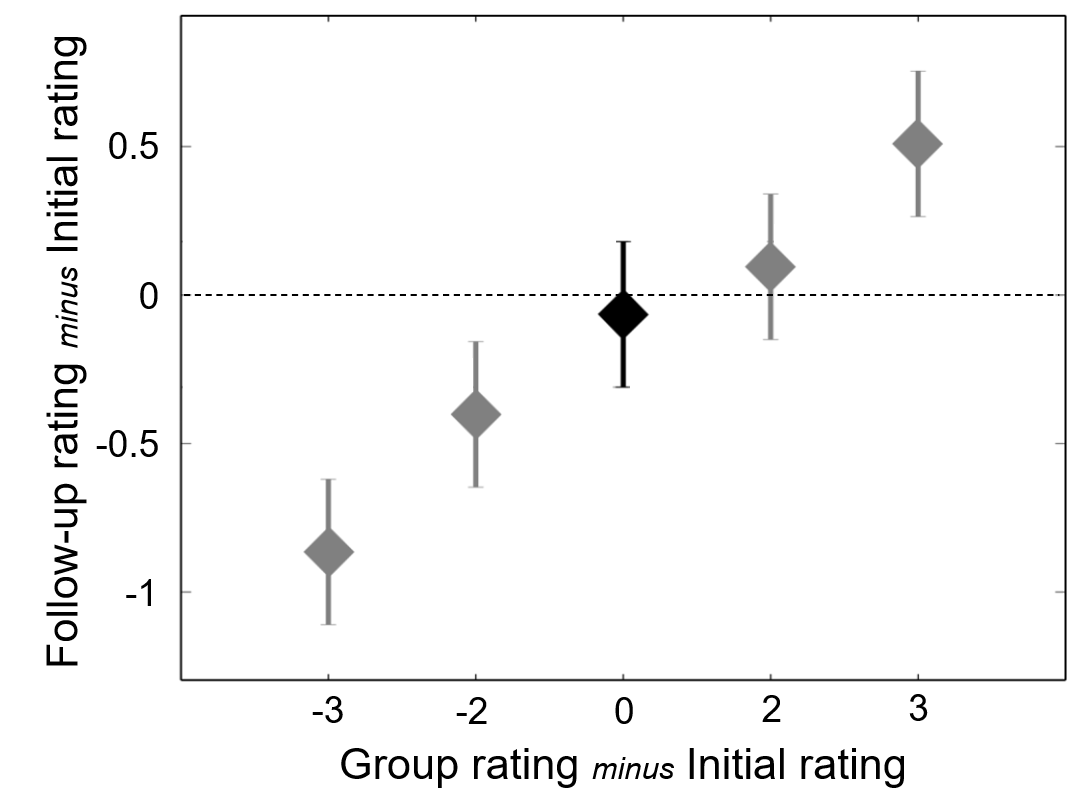


**Figure S1.** Behavioral results. The change of the mean trustworthiness rating of faces between Session 1 and Session 2 across the trials where the group rating was more negative (by 2 or 3 points), matched the subject’s rating (0), or was more positive (by 2 or 3 points). Bars indicate 95% confidence interval

Debriefing

After the Part 2 of the experiment, during a short survey, the participants answered to 3 questions:

1. Do you remember, if any faces in Session 2 were presented during Session 1?
2. How many faces were presented in both Sessions?
3. Do you remember the score you assigned to them in Session 1?

Our participants reported that some faces were presented in both Sessions. Their estimation about a number of repetitions ranged from 10 to 20 photos, and they were not able to restore the initial ratings.
Therefore, we may say that our subjects could not actively remember at least 90% of the faces.
